# The human vascular endothelial cell line HUV-EC-C harbors the integrated HHV-6B genome which remains stable in long term culture

**DOI:** 10.1007/s10616-017-0119-y

**Published:** 2017-07-28

**Authors:** Setsuko Shioda, Fumio Kasai, Midori Ozawa, Noriko Hirayama, Motonobu Satoh, Yousuke Kameoka, Ken Watanabe, Norio Shimizu, Huamin Tang, Yasuko Mori, Arihiro Kohara

**Affiliations:** 1 Japanese Collection of Research Bioresources (JCRB) Cell Bank, Laboratory of Cell Cultures, National Institutes of Biomedical Innovation, Health and Nutrition, 7-6-8 Saito-Asagi, Ibaraki, Osaka 567-0085 Japan; 2 A-CLIP Institute, Chiba, Japan; 3 Department of Virology, Medical Research Institute, Tokyo Medical and Dental University, Tokyo, Japan; 4 Division of Clinical Virology, Center for Infectious Diseases, Kobe University Graduate School of Medicine, Kobe, Japan

**Keywords:** Chromosomally integrated HHV-6, Non-tumorigenic normal cells, Genome instability

## Abstract

Human herpes virus 6 (HHV-6) is a common human pathogen that is most often detected in hematopoietic cells. Although human cells harboring chromosomally integrated HHV-6 can be generated in vitro, the availability of such cell lines originating from in vivo tissues is limited. In this study, chromosomally integrated HHV-6B has been identified in a human vascular endothelial cell line, HUV-EC-C (IFO50271), derived from normal umbilical cord tissue. Sequence analysis revealed that the viral genome was similar to the HHV-6B HST strain. FISH analysis using a HHV-6 DNA probe showed one signal in each cell, detected at the distal end of the long arm of chromosome 9. This was consistent with a digital PCR assay, validating one copy of the viral DNA. Because exposure of HUV-EC-C to chemicals did not cause viral reactivation, long term cell culture of HUV-EC-C was carried out to assess the stability of viral integration. The growth rate was altered depending on passage numbers, and morphology also changed during culture. SNP microarray profiles showed some differences between low and high passages, implying that the HUV-EC-C genome had changed during culture. However, no detectable change was observed in chromosome 9, where HHV-6B integration and the viral copy number remained unchanged. Our results suggest that integrated HHV-6B is stable in HUV-EC-C despite genome instability.

**Electronic supplementary material:**

The online version of this article (doi:10.1007/s10616-017-0119-y) contains supplementary material, which is available to authorized users.

## Introduction

A variety of human cell lines have been established from cancerous or normal cells obtained from peripheral blood or solid organs (Geraghty et al. 2014). Because of differences in tissues and individuals, characterization of each cell line is essential, especially for comparative analysis between samples. As basic quality control, cell line authentication, mycoplasma and viral tests are performed on all human cell lines in our JCRB cell bank. The possibility of viral or bacterial infection in human-origin cells cannot be excluded and raises safety concerns over their use. Although most bacteria can be eliminated from cells using antibiotics, it is difficult not only to detect viral infection in cultured cells but also to remove a viral infection from them.

Our viral test based on PCR includes human herpesvirus 6 (HHV-6), known as a cause of roseola (Yamanishi et al. 1988). After initial infection in infants, HHV-6 remains latent mainly in lymphocytes and is often found in saliva (Luppi et al. 1993). This persistent infection has the potential of reactivation, which is most common during pregnancy and is associated with fatigue. It is also known to cause critical illness in immunocompromised patients, particularly transplant recipients (Yoshikawa 2004). Different from other human herpes viruses, HHV-6 can be integrated into the host chromosome and is found in approximately 1% of the human population (Pellett et al. 2012). Integration which occurs in germinal cells leads to the transmission of the HHV-6 genome, referred to as inherited chromosomally integrated HHV-6 (Daibata et al. 1998; Gravel et al. 2015). Because the viral genome encodes telomere repeats at the both ends, this integration occurs via homologous recombination (Arbuckle et al. 2010). Consequently, HHV-6 specifically integrates into telomeres of chromosomes during latency rather than forming episomes (Arbuckle et al. 2010; Huang et al. 2014). HHV-6 is familiar to clinicians and related to various diseases, having been reported in a number of clinical cases (Hall et al. 1994, 2004; Endo et al. 2014). However, there is little human cell line established from HHV-6 integrated cells and research into HHV-6 using cell lines has so far been limited.

A human cell line established from normal umbilical vein endothelial cells, HUV-EC-C (Hoshi and McKeehan 1984), tested positive for HHV-6 in our routine viral examination. To date, the HHV-6 positive cell line is only one of over 500 human cell lines registered with our JCRB cell bank, indicating that it is exceedingly rare. This cell line has been used as an in vitro model for functional investigations of endthelial cells and toxic effects related to vascular diseases (Lindstrom et al. 1997; Park et al. 2014). In this study, the HUV-EC-C genome was characterized by chromosomally integrated HHV-6B (ciHHV-6B) at the distal end of chromosome 9q. The genome profile showed differences between low and high passages, indicating genome instability. However, the integration remained unchanged through long-term culture and upon chemical stimulation. Our results reveal that the HUV-EC-C cell line is an apparently normal cell line and the ciHHV-6B genome is stable in the human genome.

## Materials and methods

### Cells and culture conditions

A human vascular endothelial cell line derived from umbilical cord vein, HUVEC, was established by Hoshi and McKeehan (1984). The name had been changed to HUV-EC-C, which has been registered with ATCC and JCRB cell banks as CRL-1730 and IFO50271, respectively. Cells at passage 18 were deposited with our bank.

The medium used for this cell line was Ham’s F12 K (ICN Flow Laboratories (Tokyo, Japan) 10-511-20 or GIBCO (Tokyo, Japan) 21127-022) or MCDB107 with 10% FBS, 100 μg/ml heparin (Sigma (St. Louis, MO, USA) H3149) and 50 μg/ml endothelial cell growth supplement (Sigma E2759). The CS-C Complete medium Kit R (CELL SYSTEMS, DS Pharma Biomedical, Osaka, Japan) could also be used for this cell line. Cells were seeded at 3–6 × 10^5^ cells per a 100 mm dish without collagen coating, and subcultured with 0.25% trypsin-0.02% EDTA. Images were taken by a digital camera (Nikon DS-L3, Tokyo, Japan) mounted on an inverted phase contrast microscope (Olympus CKX41, Tokyo, Japan).

### DNA and RNA extraction

Genomic DNA and total RNA were extracted from HUV-EC-C cells with AllPrep DNA/RNA Mini Kit (QIAGEN, Tokyo, Japan). When extracting RNA, contaminated DNA was eliminated by digestion with RNase free DNase I (QIAGEN) at room temperature for 15 min.

### Cell line authentication

DNA samples at passages 25, 34 and 44 were amplified by the PowerPlex^®^ 16 STR System (Promega, Tokyo, Japan) and repeat numbers were determined by the ABI 3500 Genetic Analyzer.

### Flow cytometry

Expression of cell surface markers was examined at passages 27 and 49 by flow cytometry using 5 antibodies and their isotype controls listed in supplementary Table S1. Cells were stained with each antibody for 20 min at 4°C and washed twice with 4% FBS in PBS. The stained cells were fixed with 4% paraformaldehyde in PBS. The samples were run on FACSCanto flow cytometer and analyzed using Flow Jo software.

### Chromosome analysis

Cells were harvested at passage 23 after incubation with colcemid at 0.05 μg/ml for 4 h, followed by treatment with a hypotonic solution (0.075 M KCl) and three successive changes of fixative (methanol: acetic acid, 3:1). Chromosome numbers were counted on metaphases stained with Giemsa. G-banded karyotypes produced after trypsin-Giemsa staining were analyzed using the Ikaros software (Metasystems, Tokyo, Japan).

### SNP microarray

Genomic DNA samples from passages 25, 34 and 44 were examined using a high density chip, CytoScan HD array (Affymetrix, Tokyo, Japan). The data processing was performed with the Chromosome Analysis Suite software (Affymetrix).

### Sequence analysis on cancer-related genes

Target regions were amplified using the Ion AmpliSeq™ Cancer Hotspot Panel v2 (Life Technologies, Tokyo, Japan). Template DNA was prepared using the Ion PGM™ Hi-Q™ Chef Kit (Life Technologies) and sequencing was run on the Ion PGM using the Ion 316™ chip. Reads were aligned to the hg19 reference data and the analysis was carried out using the Ion Torrent Variant Caller Plugin and the Ion Reporter (Life Technologies).

### Detection of HHV-6

Detection of HHV-6 in cell lines was evaluated in their genomic DNA by the Applied Biosystems 7300 Real Time PCR system using the following primers and TaqMan probe. Forward primer (5′-GAC AAT CAC ATG CCT GGA TAA TG-3′), reverse primer (5′-TGT AAG CGT GTG GTA ATG GAC TAA-3′) and probe (5′-FAM-AGC AGC TGG CGA AAA GTG CTG TGC iowaBlack -3′). PCR product size was expected to be 176 bp, corresponding to the position 102659-102834 of the HHV-6B strain HST (AB021506.1). Human glyceraldehyde-3-phosphate dehydrogenase (GAPDH) gene was used for an amplification control, using forward primer (5′-TGT GCT CCC ACT CCT GAT TTC-3′), reverse primer (5′-CCT AGT CCC AGG GCT TTG ATT-3′) and probe (5′-FAM-AAA AGA GCT AGG AAG GAC AGG CAA CTT GGC-iowaBlack-3′). Reaction mixture was prepared in a total volume of 50 µl using Taq DNA Polymerase (Roche) on 500 ng genomic DNA for each sample. PCR was carried out by one cycle of 10 min at 95 °C, followed by 50 cycles of 15 s at 95 °C and 1 min at 60 °C.

### Viral genome analysis

**Fig. 1:**
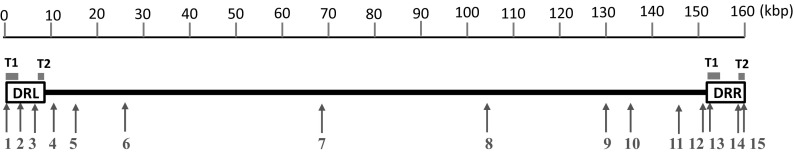
Primer positions on HHV-6B genome DNA. Each primer pair is indicated by *arrows*. Primer sequences are listed in Table S2

To reveal the contents of the HHV genome in the HUV-EC-C genome, PCR was performed on 15 regions (Fig. 1) using AmpliTaq Gold (Roche Indianapolis, IN, USA) or KOD FX (TOYOBO, Osaka, Japan) DNA polymerase. Primers were designed based on the HHV-6B HST reference sequence using a gene analysis software, Genetyx, listed in Table S2. The PCR products were run on a 2% agarose gel. A single band of the PCR products was purified by MicroSpin Columns (GE Healthcare, Tokyo, Japan) and analyzed by standard Sanger sequencing. Sequence homology of these PCR products with HHV-6B strains HST (AB021506.1), Z29 (AF157706.1) and HHV-6A U1102 (X83413.1) were analyzed by Genetyx.

### FISH mapping

Three PCR fragments (Figure S1) were labeled with Cy3, Cy5 and DNP. The metaphase preparation was treated with 70% formamide at 70°C for 2 min, and the probes were treated at 75°C for 10 min. Then, both the preparation and the probes were hybridized at 37°C overnight. After a stringent wash with standard saline citrate solution, DNP probe was detected with an anti-DNP-Alexa594 antibody. Images were captured with a Leica DMRA2 System and analyzed with Leica CW4000 FISH software. Karyotyping was analyzed with CW4000 Karyo software.

### Digital PCR

The copy number of HHV-6 in HUV-EC-C cells was examined by the QuantStudio 3D Digital PCR System (Thermo Fisher Scientific). The PCR reaction mixture was loaded onto Digital PCR 20 K Chips, and PCR was performed on the Dual Flat Block GeneAmp PCR System 9700. The PCR program consisted of one cycle of 10 min at 96 °C, followed by 39 cycles of 30 s at 98 °C and 2 min at 60 °C with a final extension of 2 min at 60 °C. TaqMan^®^ Copy Number Reference Assay RNase P was used as the standard reference for copy number analysis (Applied Biosystems, Tokyo, Japan).

### Stimulation with chemical agents

Sodium butyrate (SB) and 12-O-tetradecanoylphorbol-13-acetate (TPA) are known to induce viral lytic cycle in Burkitt’s lymphoma RAJI cells, infected with EB virus (Fang et al. 2009). RAJI cells (JCRB9012) were seeded at 5 × 10^5^ cells/ml in a 25 cm^2^ tissue culture bottle with 4 ml of 10% FBS-RPMI1640 medium. HUV-EC-C cells were seeded at 4x10^5^ cells per 100 mm dish. Cells were treated with SB and TPA, at the final concentration of 3 mM SB and 20 ng/ml TPA. At days 2, 4, and 6, cells were lysed with RLT PLUS buffer included in AllPrep DNA/RNA Mini Kit (QIAGEN). First-strand cDNA was synthesized using High Capacity cDNA Reverse Transcription Kit (Applied Biosystems). Primer sequences for the detection of BZLF-1 expressed by EB virus and U90 by HHV-6 were obtained from previous studies (Fang et al. 2009; Ihira 2012). PCR was performed using Taq DNA Polymerase (Roche) and the products were run on a 1.2% E-GEL (Invitrogen). Copy number of HHV-6 DNA was examined by digital PCR after SB and TPA treatment at different concentrations for 24 h.

## Results

### Characterisation of the HUV-EC-C cell line

#### Cell culture

**Fig. 2:**
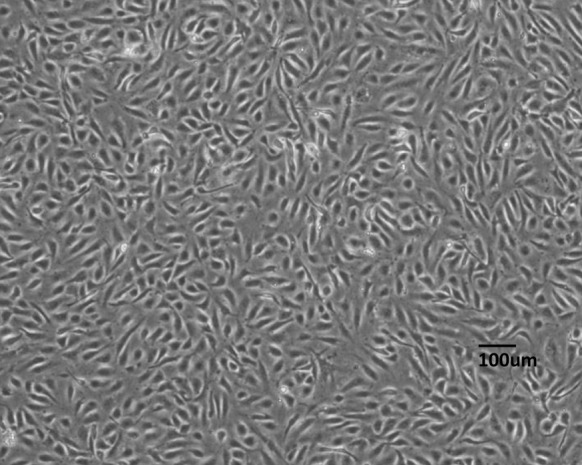
HUV-EC-C cells in confluence at passage 31. Cells were cultured for 6 days after subculturing. The morphology is typical of endothelial cells, showing space between cells. These cells do not demonstrate the cell adhesion typically observed in epithelial cells. *Scale bar* represents 100 μm

HUV-EC-C showed typical endothelial cell shape and developed a “stone paved” morphology when confluent (Figs. 2, S2). Different from epithelial cells which establish cell–cell adhesion, gaps were observed between cells. Cells were subcultured once a week and split at a 1:4 ratio. Cell density in the confluent state was approximately 2–4 × 10^4^ cells/cm^2^. The seed stock was prepared at passage 21, and a large number of stocks for distribution were prepared around passage 25. The medium necessarily required endothelial cell growth supplement and heparin. Although primary HUVEC cells were cultured on collagen coated dishes (Hoshi and McKeehan 1984), the present cell line HUV-EC-C can grow on uncoated dishes.

#### Cell proliferation

**Fig. 3:**
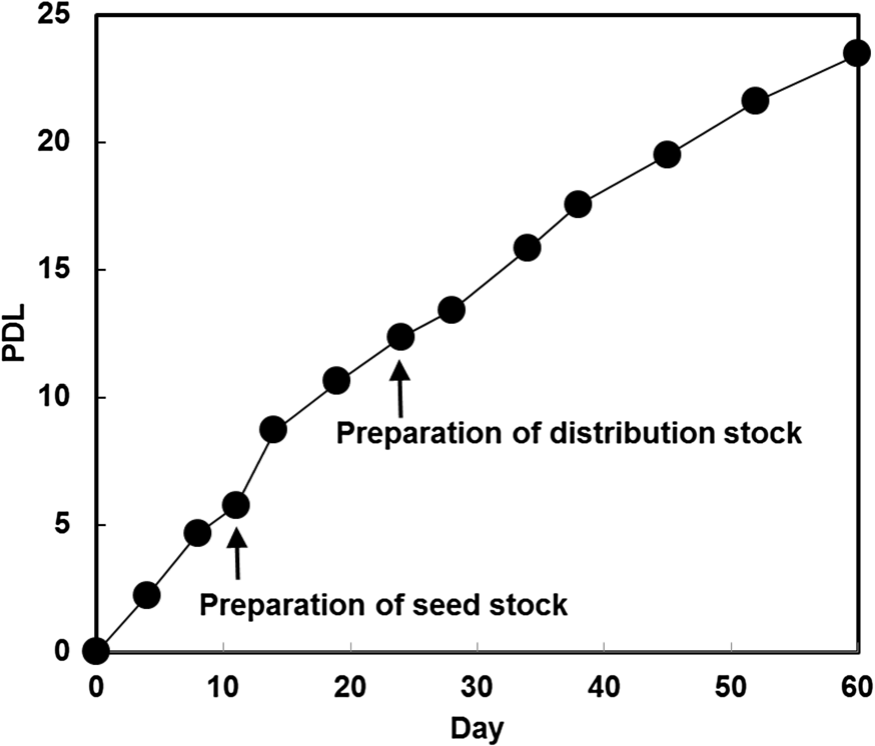
History of cultivation and growth properties of HUV-EC-C after deposition with JCRB Cell Bank. Cell culture began with cells at passage 18 and continued until passage 30. *Black circles* correspond to points of subculture

**Fig. 4:**
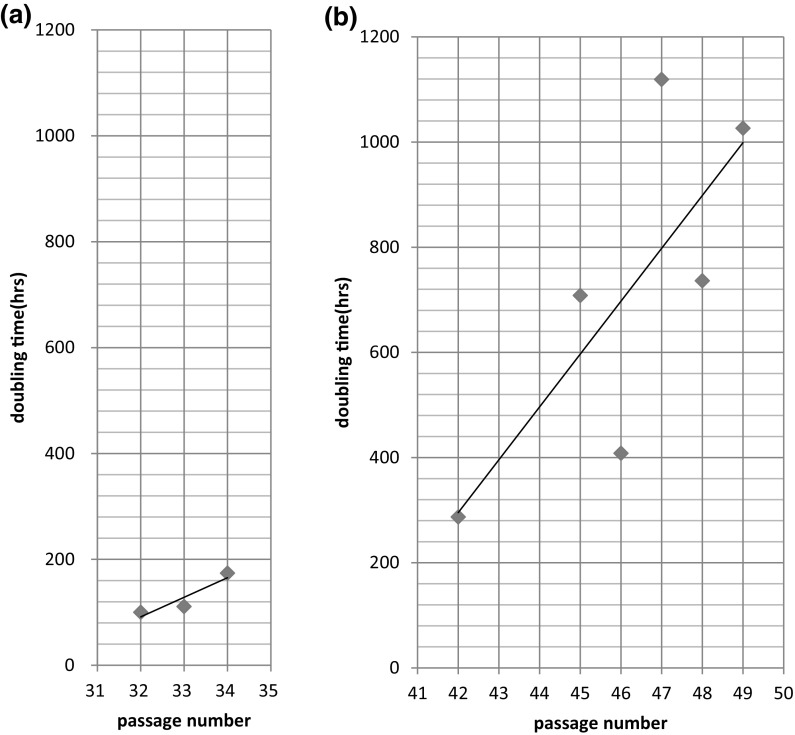
Comparison of doubling time between low passages, P32-P34 (**a**), and high passages, P42-P49 (**b**). Cells at low passages grew confluent within one week. At high passages, it took more than 2 weeks to become confluent. The trendline shows a steeper angle at higher passage numbers. This appears to demonstrate a tendency for slow growth rates, indicating that the rate of cell death is increasing, whilst the number of dividing cells is decreasing

Population doubling level (PDL) examined between passages 18 and 30 was calculated to be 23.5, shown in Fig. 3. Doubling times between passages 24 and 27, 27 and 30, 32 and 34 were estimated to be approximately 67, 84 and 100 h, respectively. After passage 40, HUV-EC-C cells became morphologically heterogeneous. Some cells became flat, large, small or multinucleated, shown in Figure S2. Cell density was decreasing, and doubling time was prolonged (Figs. 4, S3). Finally, growth halted at passage 54.

#### STR profile

STR profiles of 16 loci are shown in Table S3, confirming the same origin between IFO50271 and CRL-1730. However, changes were detected which occurred between passages 25 and 34/44 (Table S3). Two different repeat lengths were detected for D13S317 at passage 25, which became one at passages 34 and 44 by the loss of one type.

#### Cell surface markers

Flow cytometry detected the expression of vascular endothelial surface antigens, CD73 and CD105, in HUV-EC-C cells (Figure S4). CD46 and CD134 reported as cellular receptors for HHV-6 (Santoro et al. 1999; Mori et al. 2004; Tang et al. 2013) were detected and not detected, respectively (Figure S4). There was no difference in the expression of these 4 markers between passages 27 and 49.

#### Karyotyping

**Fig. 5:**
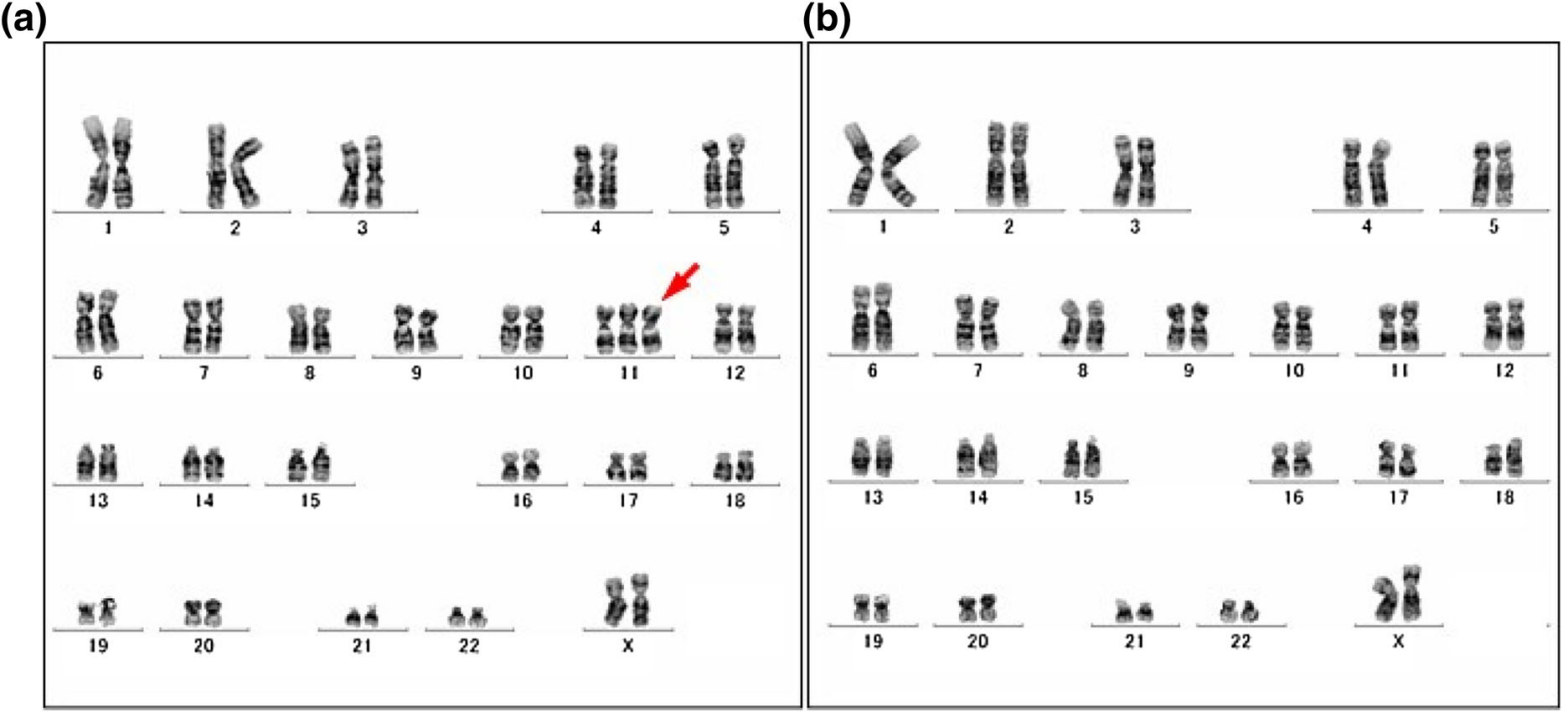
A derivative clone with 47 chromosomes of trisomy 11, indicated by an* arrow* (**a**). G-banding karyotypes of the predominant cell with 46 chromosomes, showing apparently normal female (**b**)

Chromosome analysis examined in 50 cells at passage 23 showed a normal female karyotype with a modal number of 46 chromosomes in 41 cells (Figure S5). Other karyotypes reflected 45, XX, −13 and 47, XX, +11 in 1 and 6 cells, respectively (Fig. 5).

#### Genome profile

**Fig. 6:**
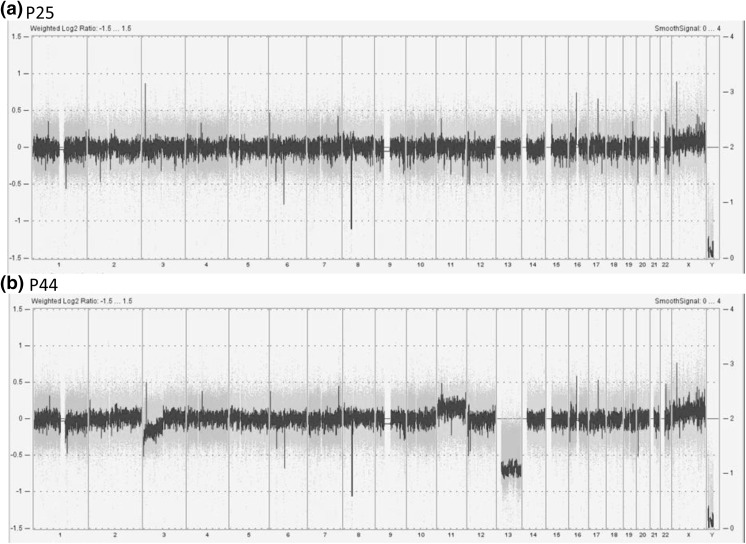
Whole genome profiles based on SNP-based microarray show differences between low (**a**) and high (**b**) passages. At passage 25, no major change is detected, however, monosomy of chromosome 13, mosaic gain of chromosome 11 and partial loss at 3p are observed at passage 44

SNP microarray revealed an apparently normal female profile at passage 25 (Fig. 6a). At passage 34, monosomy 13 and minor loss at 3p were detected (Figure S6a). These changes were also identified at passage 44, which had an additional mosaic gain of whole chromosome 11 reflecting a trisomy 11 in a small population (Fig. 6b).

Although Log2 ratio indicates a slight increase of X chromosome (Fig. 6), G-banding analysis in 50 cells at passage 23 demonstrated that both X chromosomes appeared normal (Fig. 5). Mosaic status in array profiles corresponds to heterogeneous cell population, which could be changed through cell culture. However, X chromosome profiles from 3 different passages were highly similar, implying that the gain detected in X chromosomes could be caused by polymorphism.

#### Variant profile

Within the total amplicon size of 5.67 kb covering hotspot regions of 50 major oncogenes and tumor suppressor genes, variants were detected at 15 positions located in 12 different genes, including 3 positions reported in the Catalogue of Somatic Mutations in Cancer (COSMIC) (Table S4). Missense variants were detected at 3 positions, however, these variations are registered in a SNP database, dbSNP. Frequencies were close to 50% at 9 positions and 100% at 6 positions, corresponding to heterozygous and homozygous changes. Variant frequencies were not significantly changed between different passages and no additional variant was detected. It is suggested that the loss of both RB1 and p53 functions is required for cell immortalization (Markl and Jones 1998). However, no functional mutations in these genes were detected in the HUV-EC-C, indicating that this cell line is not immortalized, and retains some aspects of a normal cell.

### Investigation of ciHHV-6 in HUV-EC-C

#### Identification of HHV-6

The real-time PCR TaqMan probe method detected HHV-6 amplified at the same Ct value as GAPDH. HHV-6 fragments covering 15 different regions were amplified from HUV-EC-C genomic DNA (Figure S7), indicating that almost the entire HHV-6 genome was integrated into the human genome. Sequence analysis of the PCR products revealed that 10 ORFs had higher homology with HHV-6B than HHV-6A (Table 1). A significant difference between HHV-6B HST and Z29 can be found in the CA repeat length in the vicinity of the HHV6B0K2-F region included in the terminal direct repeats (DRs) (Figure S8). HST strain has 12 repeats (DDBJ, ABO21506), in contrast to the absence in the Z29 strain (DDSJ, AF157706). HUV-EC-C showed 20 repeats, indicating that the ciHHV-6B would be a derivative of the HST strain.

**Table 1:** Sequence Homology of HUV-EC-C ciHHV-6 with HHV-6B strains HST(AB021506.1), Z29 (AF157706.1) and HHV-6A strain U1120(X83413.1)

Primer No.	1	2	3	4	5	6	7	8	9	10	11	12	13	14	15
ORF sequence (bp)	LT1 321	DR3 561	DR7 497	U2 638	U5 559	U14 402	U41 347	U66 144	U86 717	U89 203	U95 600	PREP 456	U98 386	DR7 798	DRHN2 425
Homology (%)															
HHV-6B HST	***98.1***	***99.1***	***98.8***	***100***	***100***	***98.3***	***98.8***	***100***	***98.7***	***98.5***	***99.7***	***99.3***	***99.2***	***99.4***	***93.7***
HHV-6B Z29	***94.6***	***97.9***	***98.6***	***99.4***	***100***	***97.8***	***98.8***	***99.3***	***98.2***	***96.1***	***99.2***	***97.4***	***94.9***	***98.7***	***90.6***
HHV-6A U1102	81.8	81.1	85.7	***95.4***	***94.5***	89.8	***94.8***	***93.7***	82.5	85.2	82.7	84.5	83.6	81.8	72.7

More than 90% homology regions are indicated by bold italic values

#### Mapping of HHV-6 by FISH

**Fig. 7:**
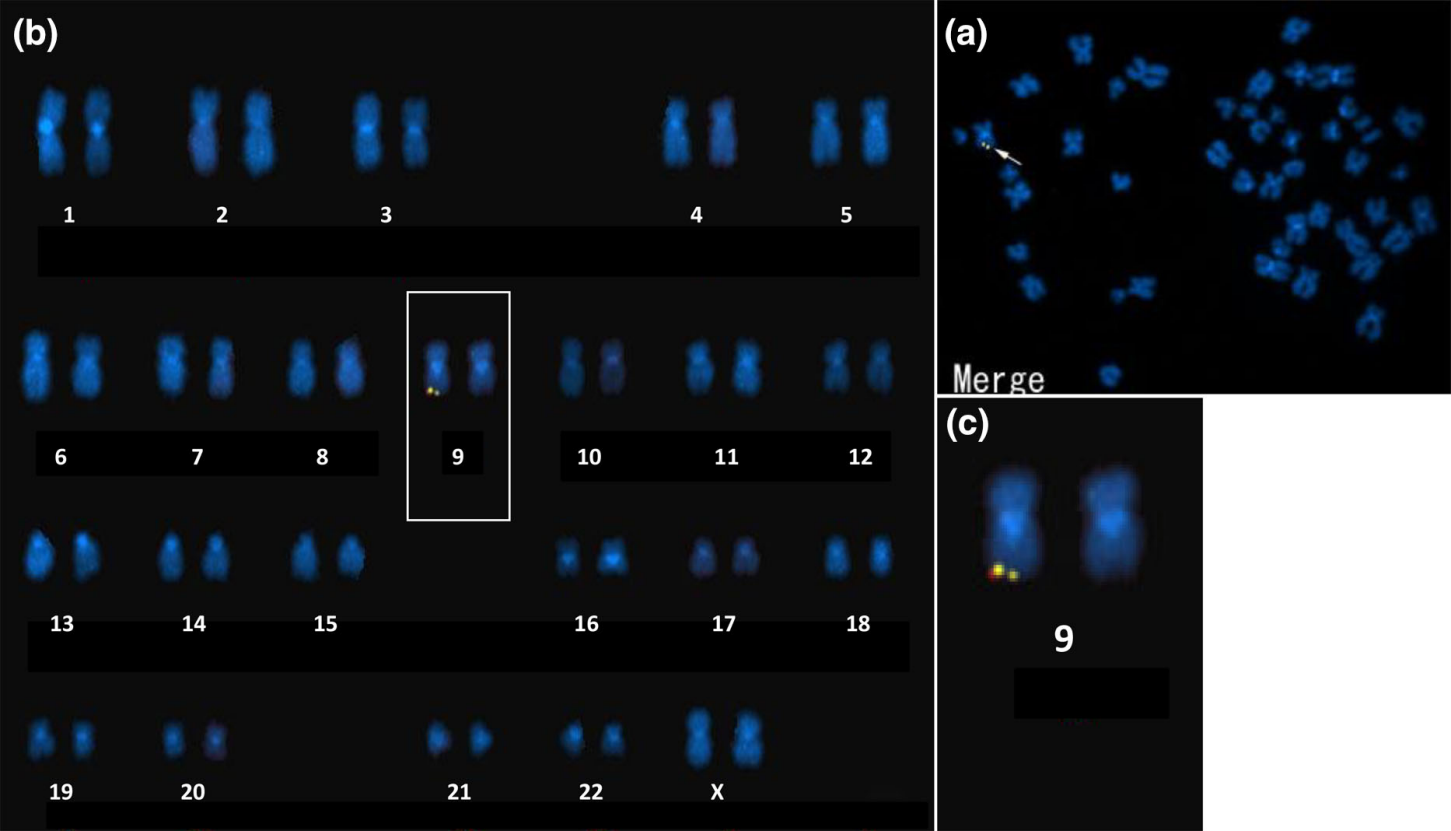
In situ hybridization of HHV-6 probes on metaphases of HUV-EC-C (**a**). Three DNA probes of HHV-6B 5-10 kbp labeled with Cy3, DNP and Cy5 hybridized to one of chromosome 9 at the distal end of the long arm (**b**), and zoomed (**c**)

To determine whether the HHV-6B viral genome was integrated into the chromosomes of HUV-EC-C cells, we designed three fluorescence in situ hybridization (FISH) probes for HHV-6B DNA (Figure S1). FISH analysis with these probes derived from clonal HHV-6B DNA fragments was performed on metaphase chromosomes from HUV-EC-C cells. The FISH analysis revealed that hybridization signals were detected at the distal end of the long arm of chromosome 9 (Fig. 7). This genomic rearrangement was not detected in the array profiles, indicating that the integration occurred in the subtelomere which was outside of the array target region (Figure S6b).

#### Copy number of HHV-6

**Fig. 8:**
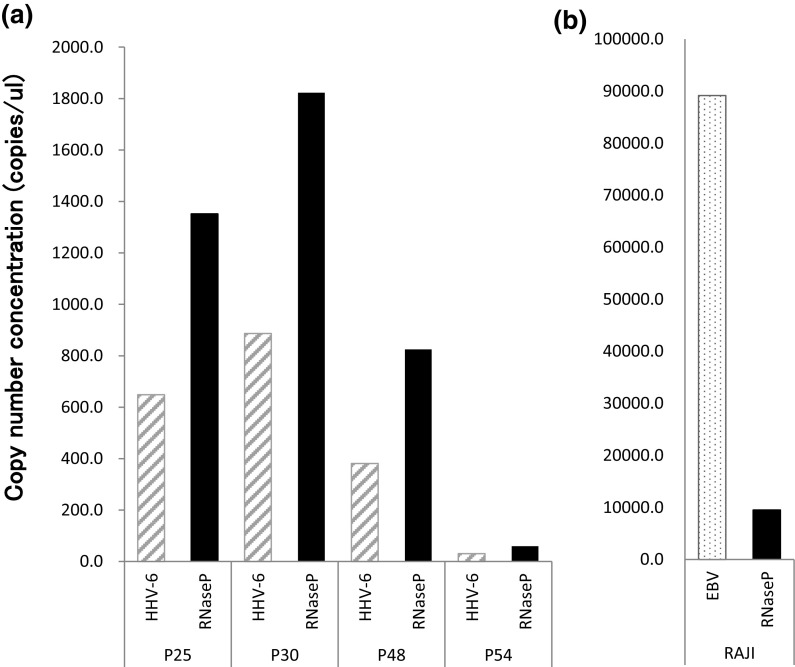
Copy number of HHV-6 DNA in HUV-EC-C (**a**). The *slash* columns indicate the copy number of U66 ORF representing HHV-6. The *stippled* column is the copy number of EBV in RAJI cells (**b**). The *black* columns are the copy number of RNase P in their hosts as a reference

The HUV-EC-C genomic DNA was compared between four different passages, 25 30, 48 and 54. As a positive control, RAJI cells, EBV harbored Burkitt’s lymphoma, were used. TaqMan^®^ Copy Number Reference Assay RNase P was run as the standard reference assay for copy number analysis. This detects the Ribonuclease P RNA component H1 (H1RNA) gene (RPPH1) on chromosome 14q11.2, which presents two copies in a diploid genome. The copy number of HHV-6 U66 was half of RNase P, and this was the same between different passages (Fig. 8). This suggests that one copy of HHV-6 virus genome is integrated in the HUV-EC-C genome.

#### Response to chemicals

**Fig. 9:**
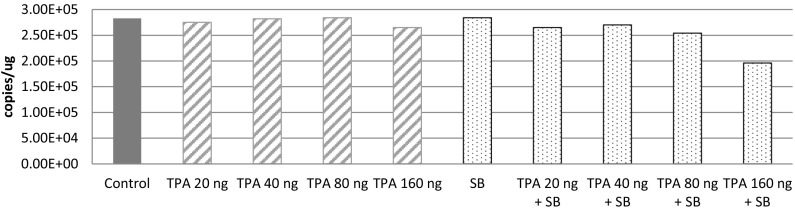
Copy number of HHV-6 DNA after chemical treatment. The copy number was assessed by digital PCR. Sample DNA was collected after treatment with various combinations of 3 mM SB and different concentration of TPA for 24 h. The copy number was not increased under these conditions

RAJI cells treated with SB and TPA became adherent and the viability was measured to be 98% at day 2 and 66% at day 6. HUV-EC-C cells remained adherent but compromised their stone paved morphology at day 2. Dead cells and debris appeared at day 4, and most cells became detached by day 6. BZLF-1 transcripts were present in the RAJI cells after the treatment, whereas they were absent in the control cells (Figure S9). However, no transcript of U90 was detected in HUV-EC-C through SB and TPA treatment, while GAPDH was expressed (Figure S10). Copy number of HHV-6 DNA was not influenced by chemical treatments (Fig. 9), suggesting that reactivation of ciHHV-6B did not occur in HUV-EC-C.

## Discussion

The HUV-EC-C cell line used in this study was not transformed (Hoshi and McKeehan 1984) and the SNP array profile at passage 25 shows apparently normal female without mutations in cancer related genes, indicating that it retains major characteristics of normal cells in the low passage. Most normal human somatic cells shorten telomeres towards senescence, although immortalized cells maintain telomere length by telomerase (Harley et al. 1990; Kiyono et al. 1998). Immortalized human endothelial cell lines established using hTERT represent stabilized telomeres (Yang et al. 1999). The growth rate of the HUV-EC-C cells gradually decreased during the serial passages and the cells stopped growing around passage 50. Because these features are commonly observed in untransformed human cell lines, the HUV-EC-C cells have finite proliferation potential due to cellular senescence without telomerase activity. A previous study using EB virus transformed lymphoblastoid cell lines describes that the HHV-6 integrated telomeres are often short and unstable, resulting in telomere fusions and chromosome instability, which lead to the viral excision (Huang et al. 2014). However, the effect of immortalization on telomere functions should be considered in the analysis of the HHV-6 integration into telomeric regions. Our results demonstrate that the HHV-6 integration is stable in the HUV-EC-C cells and the telomeric region is not altered, implying that this integration affects neither the cellular proliferation nor telomere functions.

Chromosome abnormalities, monosomy 13 and trisomy 11, have been observed in human vascular endothelial cells (Zhang et al. 2000; Anno et al. 2007). Among 50 HUV-EC-C metaphases at passage 23, chromosome analysis detected 15 abnormal karyotypes including 12 cells of trisomy 11 and one cell of monosomy 13, indicating heterogeneous cell populations. These changes were not identified in the array profile at passage 25. This discrepancy could be caused by frequency of dividing cells due to a growth advantage of abnormal cells. Because no karyotypes with both monosomy 13 and trisomy 11 existed at passage 23, these two abnormalities arose independently. However, at a later stage, all cells showed monosomy 13, suggesting that cells with trisomy 11 were eliminated through dilution while subculturing. This could demonstrate a difference in proliferation rate; cells with monosomy 13 might have a higher rate than those with trisomy 11. The array profiles show monosomy 13 at passages 34 and 44. Mosaic trisomy 11 is detected at passage 44. Cells with both monosomy 13 and trisomy 11 can be explained by duplication of chromosome 11 in a monosomy 13 cell. The duplication would be a recurrent event, implying that one of the chromosome 11 alleles in the HUV-EC-C cells was unstable.

HHV-6 genome is comprised of a large unique region (U) and two direct repeat regions, DR-L and DR-R, in the 5′ and 3′ ends, respectively (Dominguez et al. 1999). HHV-6 is classified into two variants, HHV-6A and HHV-6B, based on differences in their tropism (Mori 2009). These two variants can be distinguished by their downstream sequence. Sequence analysis of the five selected ORFs between U86 and U100 genes in the HUV-EC-C cells shows significantly higher homology to HHV-6B references. Among HHV-6B, two strains, HST and Z29, are well established as standards. The presence of the CA repeats in HUV-EC-C identifies the origin of the ciHHV-6 as a derivative of the HHV-6B HST strain.

Chromosomally integrated HHV-6 (ciHHV-6) has been defined as a condition in which the complete HHV-6 genome is integrated into the host germ line genome (Kaufer and Flamand 2014). Although ciHHV-6 is detected in various human chromosomes, the integration sites have been found specifically in telomeric regions (Morissette and Flamand 2010). It is suggested that the integration of HHV-6 is mediated by homologous recombination between telomere repeat sequence present at the end of the viral genome and human telomeres (Wallaschek et al. 2016). Once integrated, ciHHV-6 can be vertically inherited from one generation to the next (Pellett et al. 2012). Subsequently, every nucleated cell in the whole body has ciHHV-6 in its genome. Because ciHHV-6 is estimated to be approximately 1% of the normal population, there would be cell lines with ciHHV-6 derived from various tissues. However, cell lines carrying HHV-6 are largely limited to hematopoietic cells (Ablashi et al. 1989) and ciHHV-6 has been identified only in HUV-EC-C among human cell lines registered with the JCRB cell bank. We demonstrate that ciHHV-6B in HUV-EC-C is stable through long term culture, making it unlikely that ciHHV-6 spontaneously comes out from the host genome.

HHV-6 reactivation with TPA has been reported in a latently infected astrocytoma cell line (Yoshikawa et al. 2002). Reactivation of ciHHV-6 has been also observed in immunocompromised patients and shows the possibility of the production of viral transcripts and proteins (Endo et al. 2014; Miura et al. 2015). However, reactivation of HHV-6 did not occur in the HUV-EC-C cells under drug treatment. The SNP array profiles of chromosome 9 show no changes through long term cell culture, indicating that the viral genome had tightly integrated into telomeric region and this has become stable. Instability of telomeric regions in the human genome may lead to reactivation of the viral genome.

Our analyses consisting of cytogenetic, sequencing and flow cytometry approaches correspond to characterization of cell lines at cellular and molecular levels. Curated reference profiles generated based on quality-controlled cell lines can be replenishable resources for their future use. STR profiles have been established for human cell line authentication, however, they are not always completely identical between cell lines carrying the same name depending on their culture history, number of passages or technical differences between laboratories (Freedman et al. 2015). This reflects genome instability in cell lines frequently observed during culture, which is an unavoidable consequence of cell culture (Kasai et al. 2016). In this study, HUV-EC-C is characterized by an integration of HHV-6B as its universal feature. Although genome instability occurred during culture, the rearrangement caused by the virus remains unchanged through long term cell culture. Our observations suggest that ciHHV-6B in HUV-EC-C is highly stable and can be conserved through generation.

## Electronic supplementary material

Below is the link to the electronic supplementary material.

Supplementary material 1 (PDF 1130 kb)
