# Supplementary material for: The human vascular endothelial cell line HUV-EC-C harbors the integrated HHV-6B genome which remains stable in long term culture
Source: Cytotechnology. 2017 Jul 28;70(1):141–52. doi: 10.1007/s10616-017-0119-y (PMC5809643; doi:10.1007/s10616-017-0119-y)
Supplement: Supplementary file 1 — Supplementary material 1 (PDF 1130 kb) [file 10616_2017_119_MOESM1_ESM.pdf]

Figure S1

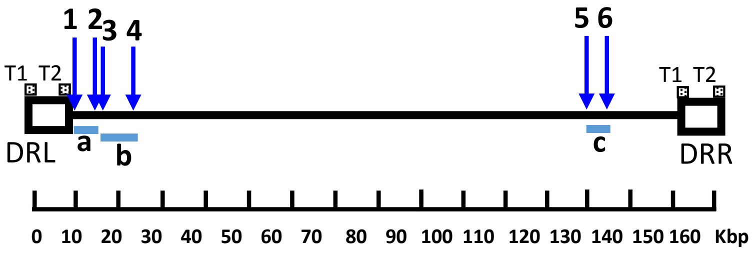

Positions of 3 DNA fragments (a, b, and c) for FISH probes in the HHV-6B genome. The blue arrows indicate the position of primer sets, 1: HHV6B9K-F, 2: HHV6BU5-R, 3: HHV6BU5-F, 4: HHV6BU14-R, 5: HHV6BU86-R, and 6: HHV6B135K-R. Primer sequences are shown in Table S2. The terminal direct repeats (DRL and DRR) are boxed, and telomeric repeat regions (T1, T2) are indicated by squares. The DNA fragments for FISH probes do not include telomeric sequence.

Figure S2

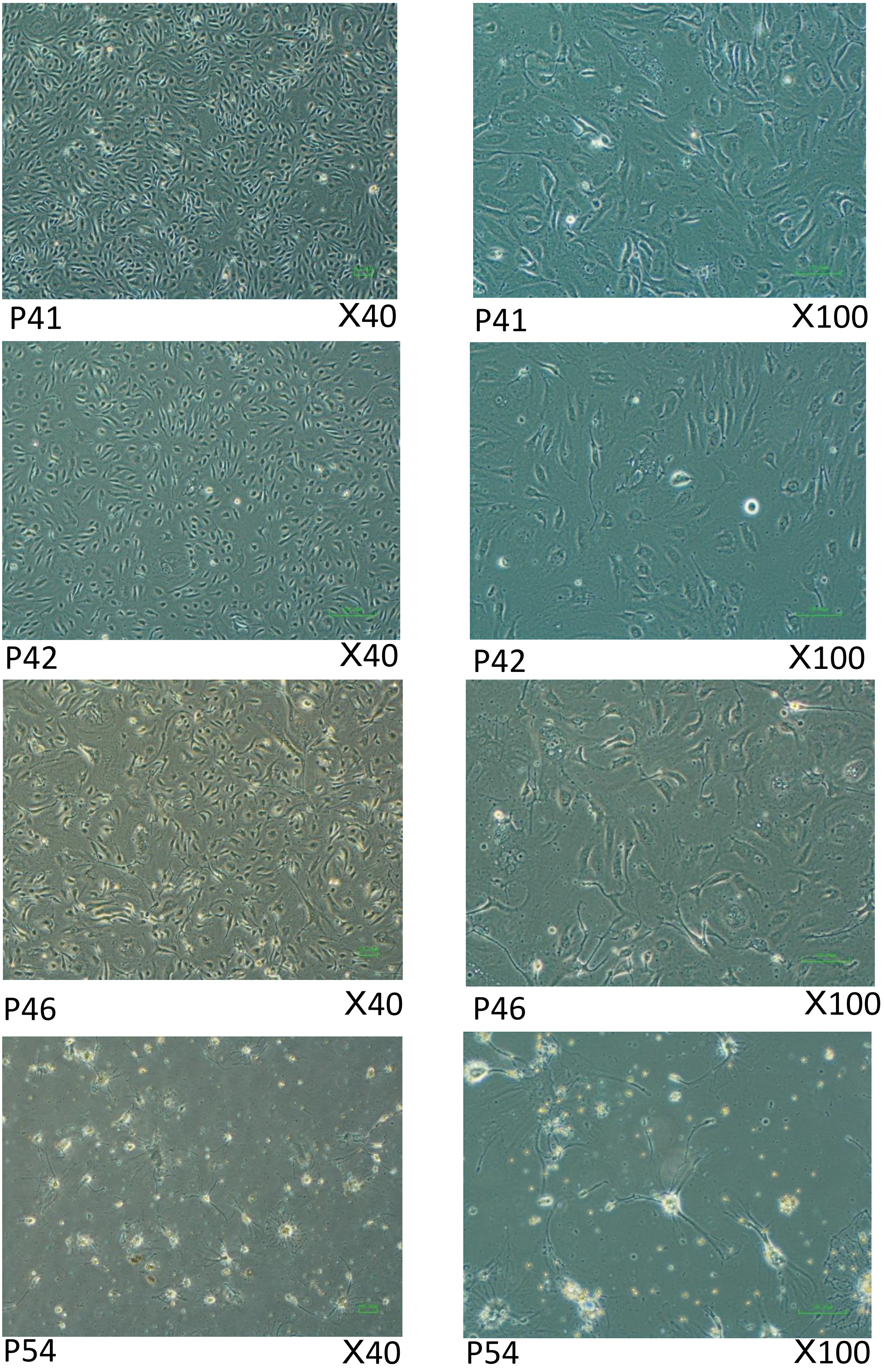

HUV-EC-C cell morphology during long term culture. At passages 41 and 42, cells remain the same density at low passages. At passage 46, the cell density was decreased and senescent cells were observed (x100). At passage p54, many round cells appeared and most cells became senescent.

Figure S3

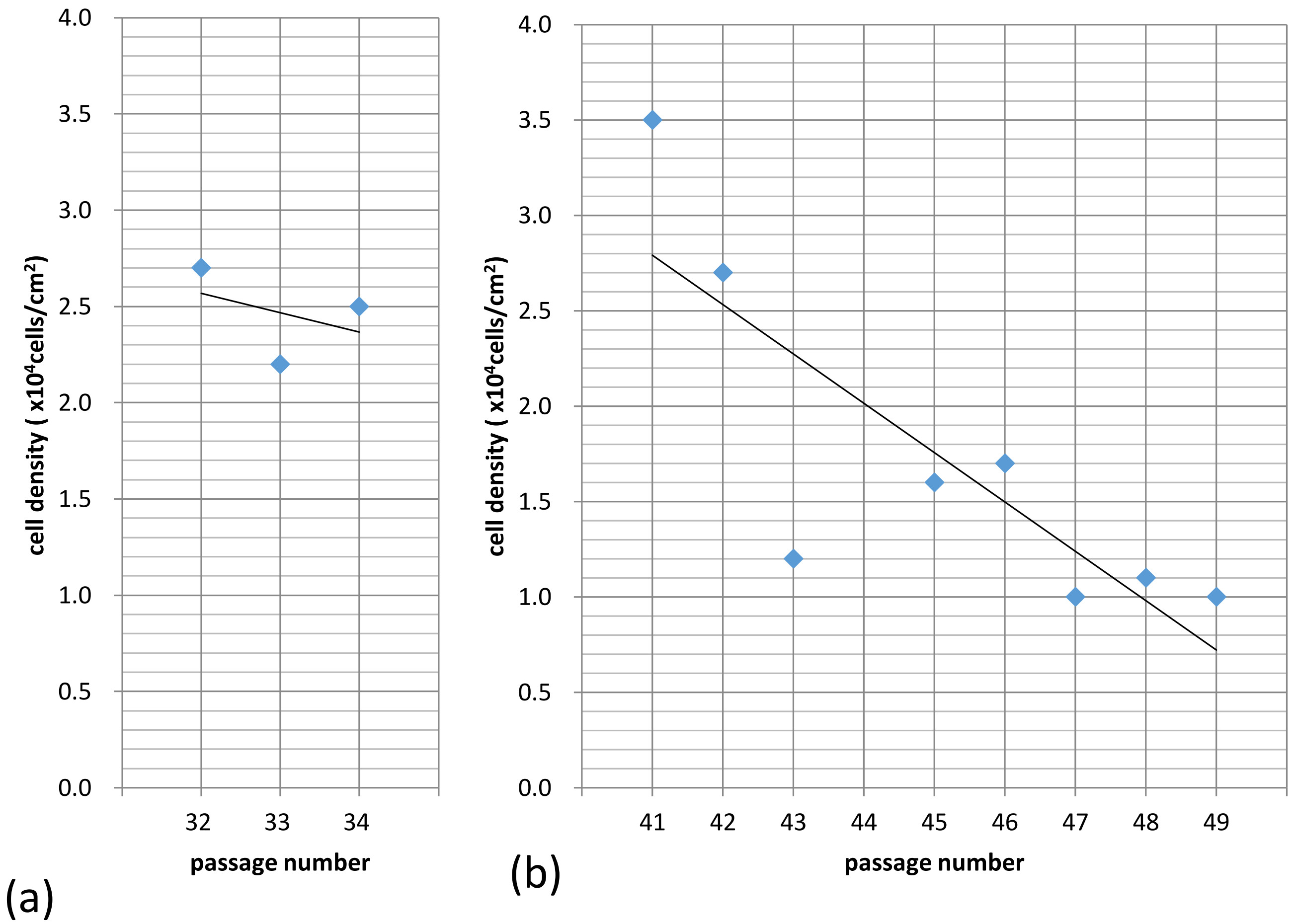

Cell density at low passages, P31-P34 (a), and high passages, P40-P49 (b). At low passages, cell density showed around 1.5 $x10^6$ cells per 100 mm dish, but it dropped to under $1.0x10^6$ cells at high passage, and significantly decreased after passage 43. The steep trendline at the high passages indicates a decrease in the growth rate.

Figure S4

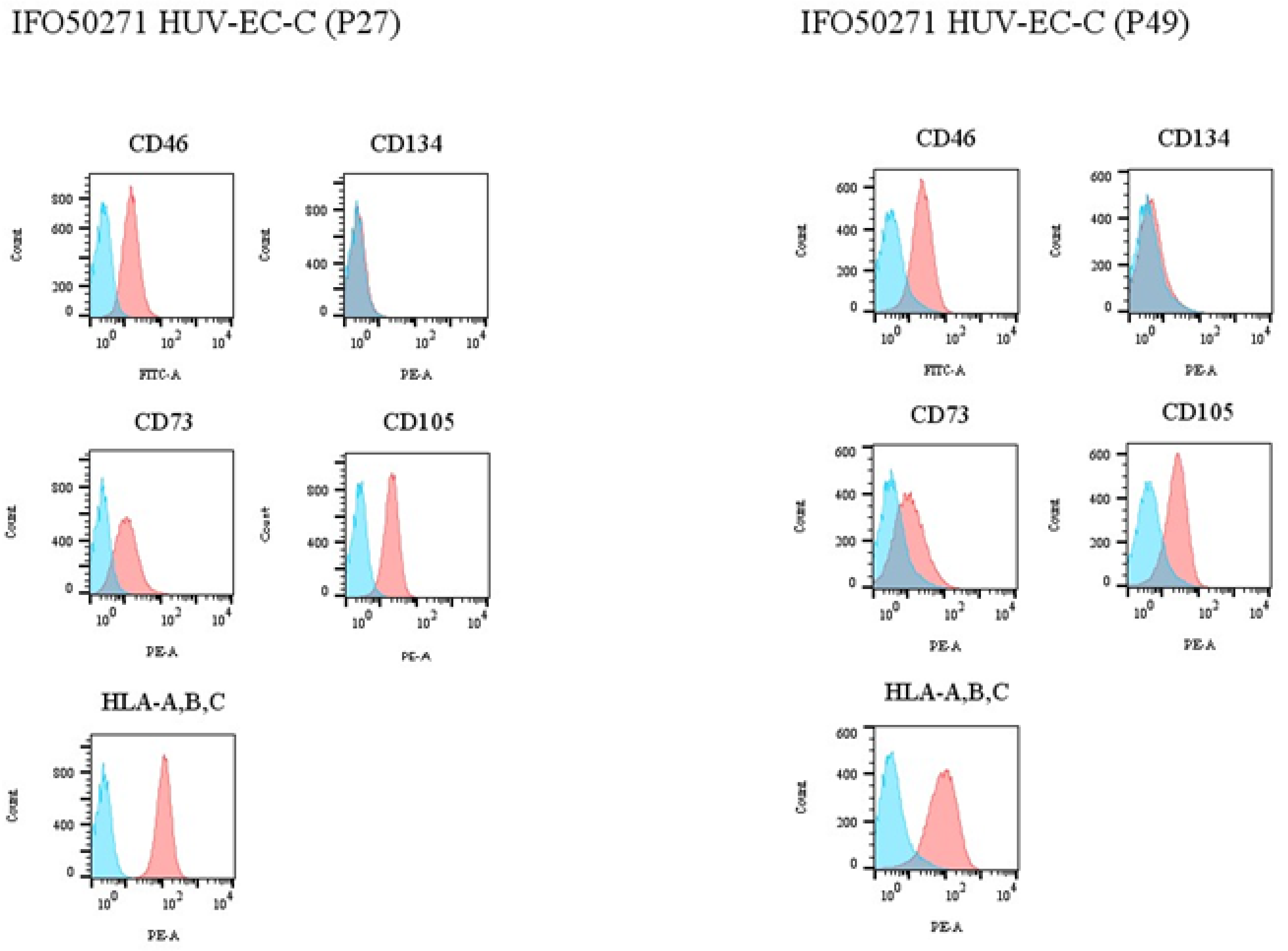

Flow cytometry analysis of expression of cell surface antigen molecules on HUV-EC-C. Profiles between passages 27 and 49 are similar, indicating that cells retain the same characteristics during long term cell culture. HLA-A, B, C were used as positive controls.

Figure S5

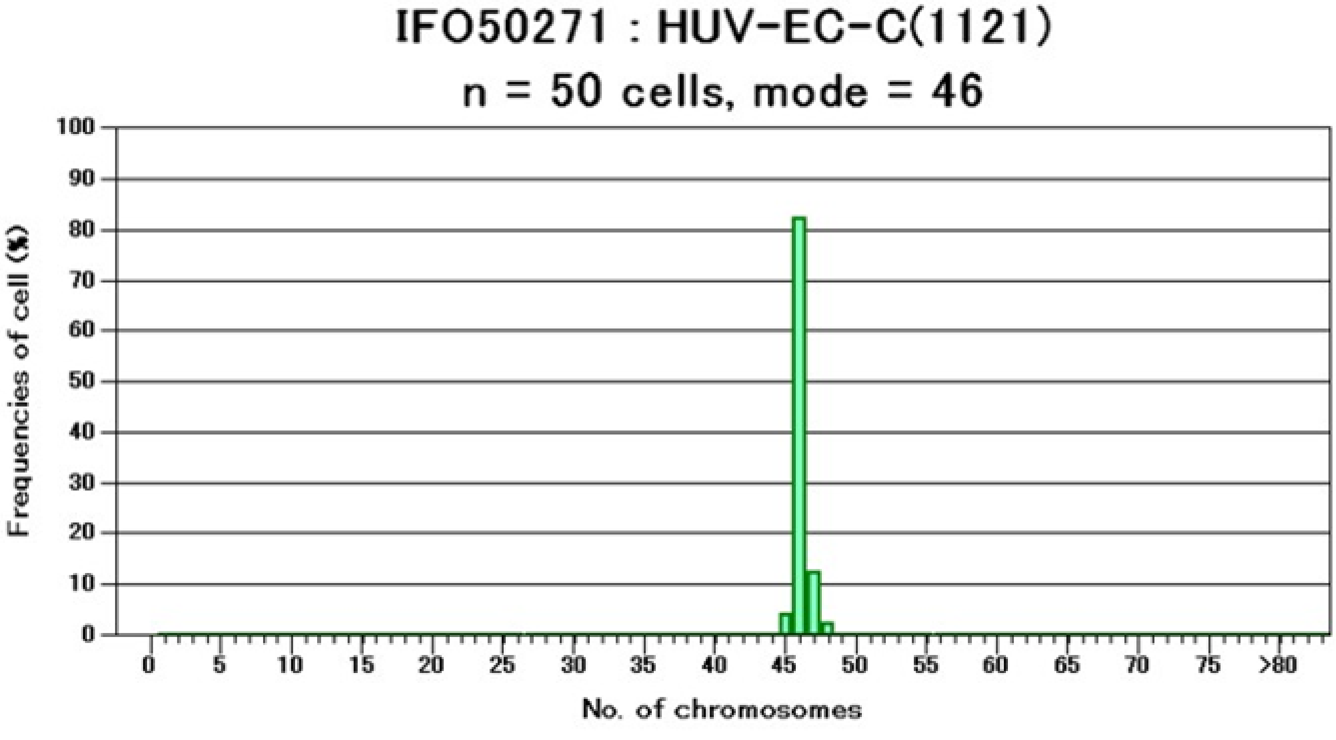

Chromosome number determined in Giemsa-stained metaphases, showing the modal number of 46 chromosomes.

Figure S6

(a)

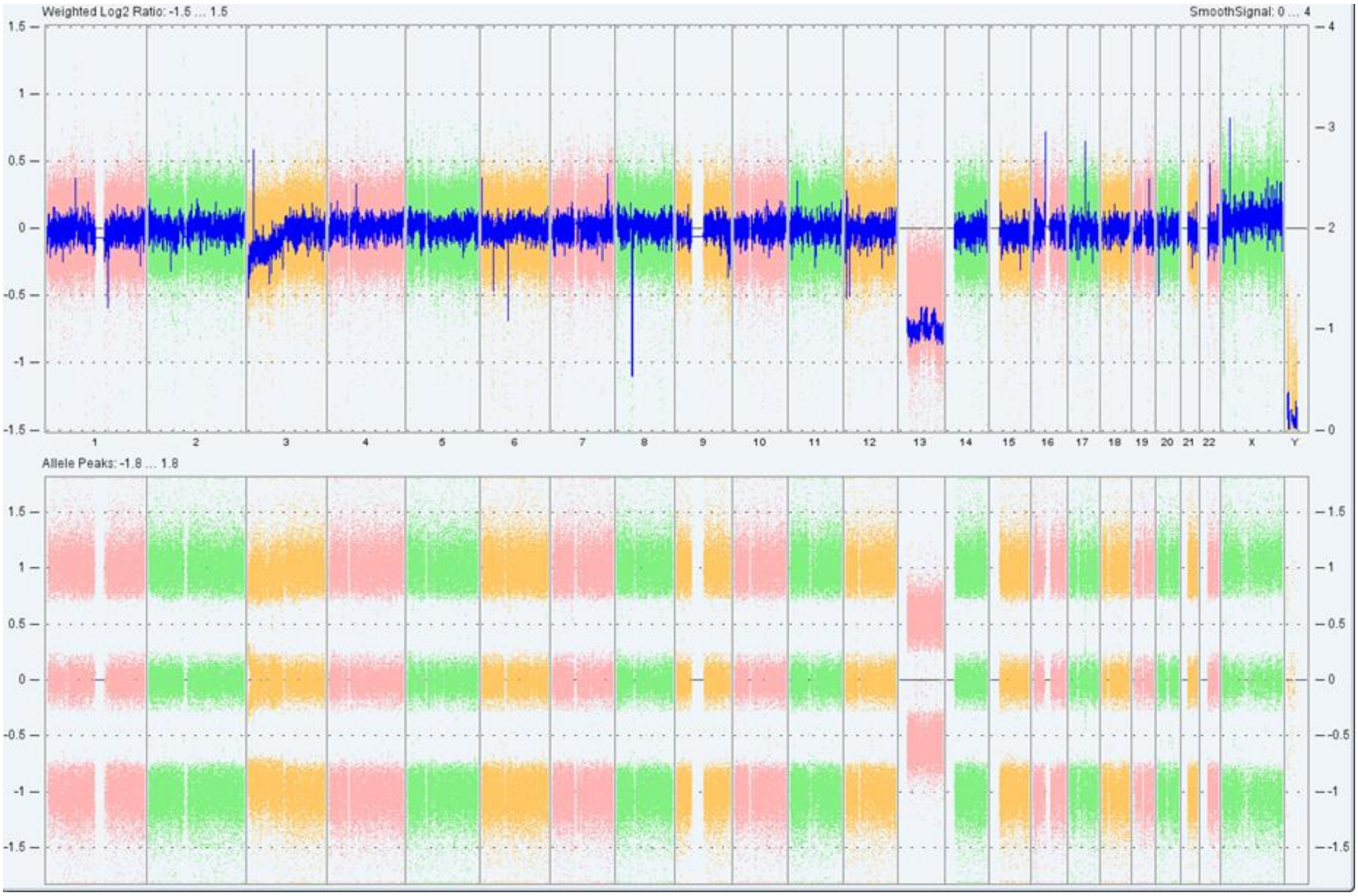

(b)

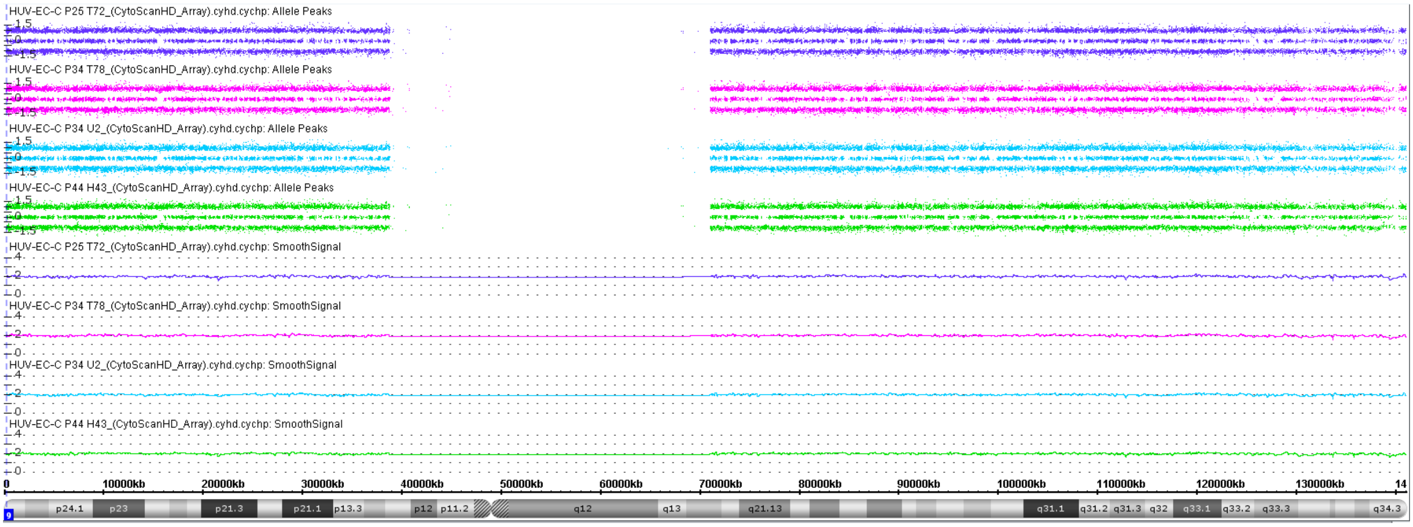

SNP-based microarray profiles at passage 34, showing monosomy of chromosome 13 and partial loss at 3p (a). SNP-based microarray profiles of chromosome 9 show no changes in copy numbers and allele peaks (b).

Figure S7

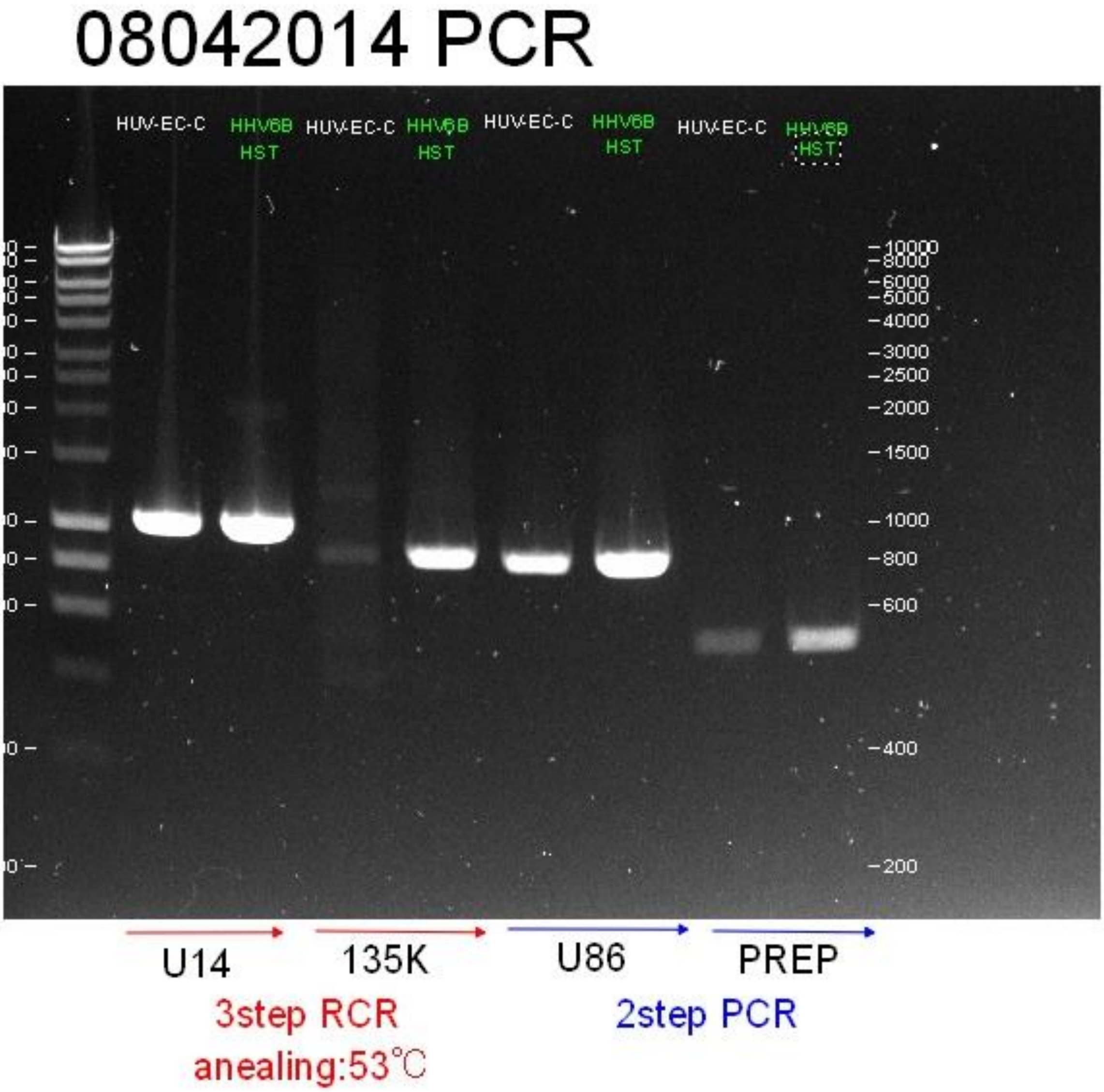

Comparison of PCR product size amplified from HUV-EC-C ci-HHV-6B and HHV-6B strain HST proliferated in cord blood mononuclear cells. Each pair of PCR products from ciHHV-6B and HHV-6B strain HST were the same size on 1.2% agarose gel.

Figure S8

```
     HST  CATCACCCCACCCGCTGCCCCCCCCC-----------------ACACAC
          **************************                 ******
HUV-EC-C  CATCACCCCACCCGCTGCCCCCCCCCCACACACACACACACACACACAC
          ******** ************ *** **  * * *
     Z29  CATCACCCAACCCGCTGCCCCACCCACATCCCCCC--------------

     HST  ACACACACACACACACACCGCCACCGCTACCACCACCTCTGAACTTC
          ***********************************************
HUV-EC-C  ACACACACACACACACACCGCCACCGCTACCACCACCTCTGAACTTC
                         ****************  **************
     Z29  ---------------CACCGCCACCGCTACCGTCACCTCTGAACTTC

     HST  ACCTTTTCCCTCCATCTCGCCCCACTTCTCTCTACA
          ***********************************
HUV-EC-C  ACCTTTTCCCTCCATCTCGCCCCACTTCTCTCTACA
          ******* ***************************
     Z29  ACCTTTTTCCTCCATCTCGCCCCACTTCTCTCTACA
```

Nucleotide sequence homology between HHV-6B strains HST, Z29 and ciHHV-6B in the HUV-EC-C genome. T1 region of the HST strain is compared with PCR products amplified using a primer set of HHV6B0K2-F and HHV6B152K2-R. HHV-6 integrated in HUV-EC-C has 20 copies of the (CA) repeat sequence, though the HST strain has 12 copies. Sequence data of strains HST and Z29 are used from the genome database, ABO21506 and AF157706, respectively.

Figure S9

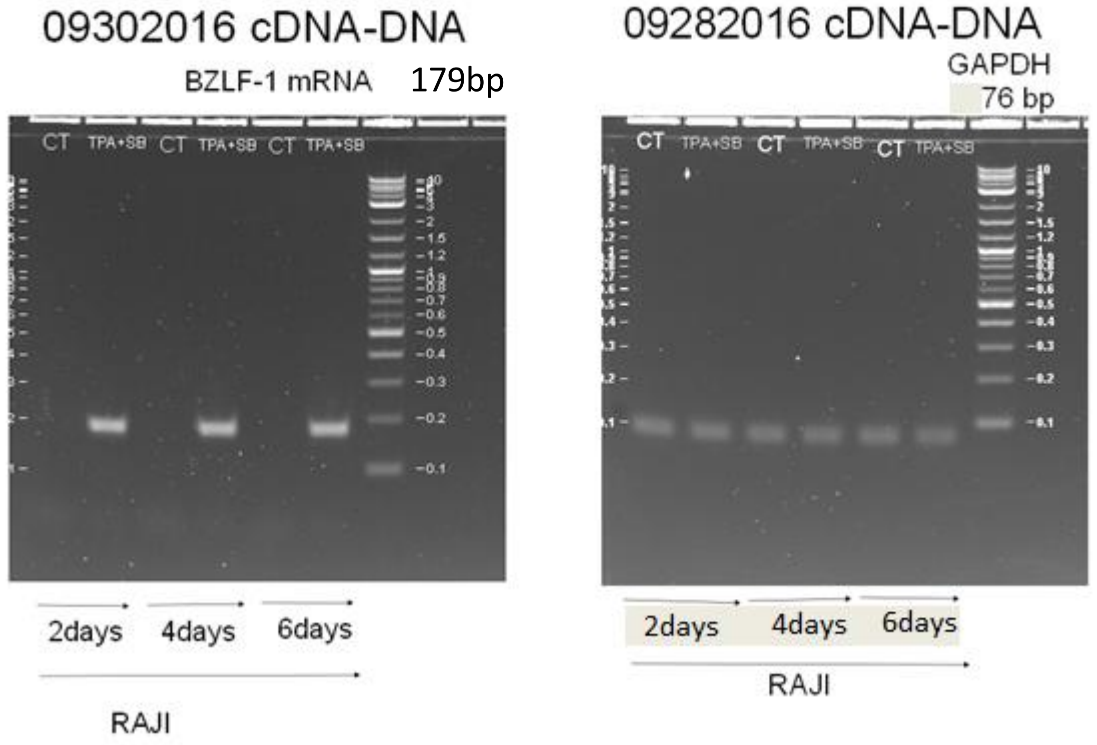

Detection of BZLF-1 and GAPDH transcriptome from total RNA extracted from both chemical treated and untreated RAJI cells. The transcriptome of mRNA BZLF-1(179 bp), was detected from total RNA extracted from RAJI cell treated with TPA and SB. Sample was harvested 2 days, 4 days and 6 days after treatment. No transcriptome was detected from untreated cells. GAPDH transcriptome (76bp) was detected in all samples. Reactivation was detected in chemical treated RAJI cells.

Figure S10

(a)

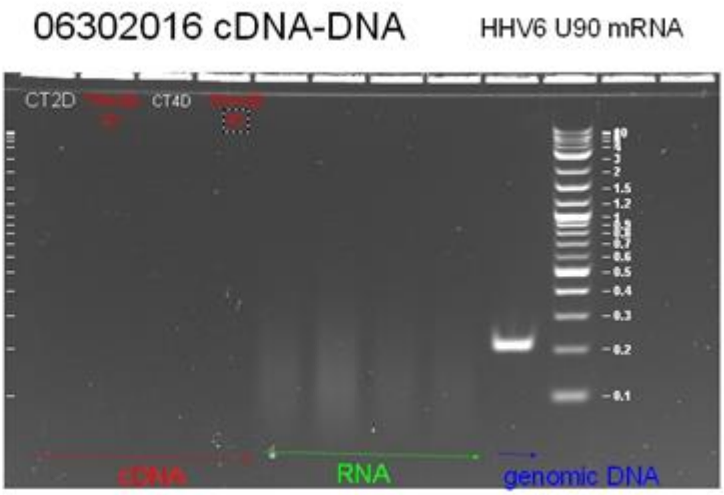

(b)

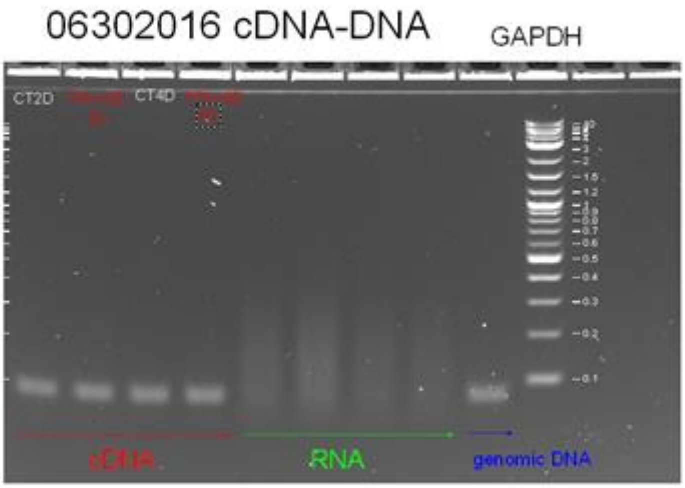

Detection of HHV-6 U90 and GAPDH transcriptome in total RNA extracted from chemical treated HUV-EC-C cells and controls. Controls were performed on genomic DNA samples. No PCR product was detected from total RNA samples, showing no contamination of genomic DNA. Although GAPDH (76bp) was detected in cDNA samples, no PCR product appeared for U90.

Table S1

Cell surface markers used for flow cytometry

cell surface antigen	Isotype	fluorescent labeling	manufacturer, cat. No.	Isotype control	fluorescent labeling	manufacturer, cat. No.
CD46	mouse IgG1	FITC	Abcam, ab28068	mouse IgG1/mouse IgG2a	FITC/RPE	Beckman coulter, A10974
CD73	mouse IgG1	RPE	BD Bioscience, 550257	mouse IgG1	RPE	DAKO, X0928
CD105	mouse IgG3	RPE	Beckman coulter, A07414	mouse IgG3	RPE	SouthernBiotech, 0105-09
CD134	mouse IgG1	RPE	Beckman coulter, A24986	mouse IgG1	RPE	DAKO, X0928
HLA-A,B,C	mouse IgG2a	RPE	DAKO, R7000	mouse IgG1/mouse IgG2a	FITC/RPE	Beckman coulter, A10974

Table S2

Primer sequence and the positions in the HHV-6B strain HST genome

No.	Name	Sequence	Position (HH-6B HST)	
1	HHV6-0K2-F	TAACACTACCCCTCTTTCAACCACTCAC	396 - 423	153438 - 153465
	HHV6-0K2-R	TGCAGTGTGTCGGCTCAGAAAC	1054 - 1033	154096 - 154075
2	HHV6-3K-F	AGTTCACACAGATGCAACC	3520 - 3538	156562 - 156580
	HHV6-3K-R	ATACTTTCGGTTTCAGGTACAG	4154 - 4133	157196 - 157175
3	HHV6-6K-F	AATTACTAGAGACCGTGCG	6738 - 6756	159780 - 159798
	HHV6-6K-R	GTATATCGCATCCTTACGTCT	7272 - 7252	160314 - 160294
4	HHV6-9K-F	GAGCAGAGGTAAAGTAACGC	9809 - 9828,	
	HHV6-9K-R	CCCTTCTTCGTGCTCTTTC	10496 - 10478	
5	U5-F	AGTCGGATCCTTTATGCTCCTGTGTGGTTGTGTC	14995 - 14971	
	U5-R	AGTCCTCGAGCATTTCCATGAACCGAGTCTCGTC	14389 - 14412	
6	U14=F	CATGGATCCATGGAAGGGTCGAAGACATTC	24136 - 24156	
	U14-R	CATGAATTCAATCCTCGAACTCTATTAGGT	25164 - 25145	
7	U41-F	AGTCGGTACCTGTATTTGTGAATGTTTTGTCAC	69335 - 69312	
	U41-R	AGTCAAGCTTACATAATCGAATAAGATCACCATGATC	68251 - 68277	
*8	HHV6U66-F	GACAATCACATGCCTGGATAATG	102812 - 102834	
	HHV6U66-R	TGTAAGCGTGTGGTAATGGACTAA	102659 - 102682	
9	U86-F	AGTCGGATCCCAGCAAGTTTCAACGTCTCCTGAC	130889 - 130866	
	U86-R	AGTCCTCGAGATTTGTATTGGCCTTCTTCAGAGC	130036 - 130060	
10	HHV6-135K-F	ATTTCCTGTTCCCTGCG	135003 - 135019	
	HHV6-135K-R	AAGTCTCTATATTCACCACCAG	135902 - 135881	
11	U95-F	AGTCGAATTCTCCATGTTTGCAGTGGAC	146312 - 146335	
	U95-R	AGTCGTCGACTAATTATACCCCAATTTCTCCTCTGAC	147091 - 147067	
12	PREP-F	ACCGCTAGCATCCGCAGCACCATCTTTGT	149606 - 149587	
	PREP-R	ACCAGATCTGCCACGAAATGCATAATTAT	149080 - 149099	
13	HHV6-150K-F	CTCAGTTTCGTACAGTGCA	150039 - 150057	
	HHV6-150K-R	CATATTGAAGTCTAAACCTGCC	150565 - 150544	
14	HHV6-159KL-F	TTCGTAAGCTCGTATCTGACCCACTCCCCGCTCGAC	6425 - 6460	159467 - 159502
	HHV6-159KL-R	TCTTCCTCCGGTCATGTCTCTGGCCGTATATCGCATCCTT	7297 - 7258	160339 - 160300
15	HHV6-160K-F	AGGTTCAAAGATAGACAGTG	7809 - 7828	160851 - 160870
	HHV6-160K-R	TTAGGGTTATAGATTGCGC	8303 - 8285	161345 - 161327

*8: This primer pair is used for a routine viral examination.

5, 6, 7, 11, 12: Tag sequence is added to the 5' ends.

Table S3

STR profiles of 16 loci.

	TPOX	D3S1358	FGA	D5S818	CSF1PO	D7S820	D8S1179	TH01	vWA	D13S317	Penta E	D16S539	D18S51	D21S11	Penta D	AMEL
	2p25.3	3p21.31	4q31.3	5q23.2	5q32	7q21.11	8q24.13	11p15.5	12p13.31	13q31.1	15q26.2	16q24.1	18q21.3	21q21.1	21q22.3	Xp22.2
P25	8, 11	16	21, 23	11, 12	11, 12	8, 12	14, 16	6, 9.3	16	9, 11	7, 13	11, 12	13, 17	28, 31	12, 13	X
P34	8, 11	16	21, 23	11, 12	11, 12	8, 12	14, 16	6, 9.3	16	11	7, 13	11, 12	13, 17	28, 31	12, 13	X
P44	8, 11	16	21, 23	11, 12	11, 12	8, 12	14, 16	6, 9.3	16	11	7, 13	11, 12	13, 17	28, 31	12, 13	X

Table S4

Variant profiles based on AmpliSeq™ Cancer Hotspot Panel

Chrom	Position	Ref	Variant	Allele Call	Type	Allele Name	Gene ID	Exon	Coding	Amino Acid Change	P25	P34	P44
chr4	1807894	G	A	Homozygous	SNP	---	FGFR3				100.0	100.0	100.0
chr4	55141055	A	G	Homozygous	SNP	---	PDGFRA				100.0	100.0	100.0
chr4	55593481	A	G	Heterozygous	SNP	COSM21983	KIT				50.6	48.7	50.5
chr4	55962546	-	G	Heterozygous	INS	---	KDR				51.9	52.9	52.7
chr4	55972974	T	A	Heterozygous	SNP	---	KDR	11	c.1416A>T	p.Gln472His	51.1	48.2	47.7
chr4	55980239	C	T	Homozygous	SNP	---	KDR				100.0	100.0	100.0
chr5	112175770	G	A	Heterozygous	SNP	---	APC				51.2	50.1	48.0
chr5	149433596	TG	GA	Homozygous	MNP	---	CSF1R				100.0	100.0	100.0
chr7	55249063	G	A	Heterozygous	SNP	---	EGFR				50.2	51.0	47.0
chr7	116339672	C	T	Heterozygous	SNP	---	MET				51.7	48.7	54.0
chr7	116340262	A	G	Heterozygous	SNP	COSM710	MET	2	c.1124A>G	p.Asn375Ser	49.4	48.1	50.1
chr10	43613843	G	T	Heterozygous	SNP	---	RET				50.7	49.7	51.0
chr11	534242	A	G	Heterozygous	SNP	COSM249860	HRAS				53.0	49.5	53.8
chr13	28610183	A	G	Homozygous	SNP	---	FLT3				100.0	100.0	100.0
chr17	7579472	G	C	Homozygous	SNP	---	TP53	4	c.215C>G	p.Pro72Arg	100.0	100.0	98.9
